# Supplementary material for: Far-red and sensitive sensor for monitoring real time H2O2 dynamics with subcellular resolution and in multi-parametric imaging applications
Source: Res Sq. 2024 Apr 17:rs.3.rs-3974015. Preprint. [Version 1] doi: 10.21203/rs.3.rs-3974015/v1 (PMC11065073; doi:10.21203/rs.3.rs-3974015/v1)
Supplement: Supplement 1 [file NIHPPrs3974015v1-supplement-1.pdf]

Supplementary Files

This is a list of supplementary files associated with this preprint. Click to download.

- NCBoROSHTmanuscriptsupplementaryfigures.pdf
